# On the Effect of Heterophilic Antibodies on Serum Levels of Cardiac Troponins: A Brief Descriptive Review

**DOI:** 10.3390/life12081114

**Published:** 2022-07-24

**Authors:** Aleksey Michailovich Chaulin

**Affiliations:** 1 Department of Cardiology and Cardiovascular Surgery, Samara State Medical University, 443099 Samara, Russia; a.m.chaulin@samsmu.ru; Tel.: +7-(927)-770-25-87; 2 Department of Histology and Embryology, Samara State Medical University, 443099 Samara, Russia

**Keywords:** heterophile antibodies, cardiac troponins, false-positive results, cardiovascular diseases, myocardial infarction

## Abstract

Serum levels of cardiac troponins can be increased both with myocardial damage and in the absence of myocardial damage. In the second case, this is due to the influence of false-positive factors, among which heterophilic antibodies play a significant role. Understanding the causes of the formation of heterophilic antibodies, the features and mechanisms of their effect on serum levels of cardiac troponins, is an important condition for interpreting a false-positive result due to the influence of heterophilic antibodies. This brief, descriptive review presents the causes of heterophilic-antibodies formation and discusses their effect on serum levels of cardiac troponins.

## 1. Introduction

Cardiospecific troponins T and I (cTnT and cTnI) are, undoubtedly, considered the most effective biomarkers of myocardial infarction (MI), due to the two main criteria of an ideal biomarker: high sensitivity and specificity [1,2,3,4]. At the same time, it is known that, apart from myocardium, troponins are expressed in skeletal-muscle tissue and the walls of venae cavae and pulmonary veins [5,6,7,8].

From the moment the first immunoassays were invented, the methods for detection of cTnT and cTnI in serum have been refined, which led to a revolution in MI diagnostics. First of all, their sensitivity significantly increased, while the limit of detection (LoD) or the minimum detectable concentration (MDC) of the first prototypes was about 100–500 ng/L, in modern immunoassays it can be even less than 1 ng/L [1,9,10]. Therefore, it became possible to detect the extremely low concentration of troponin equal to 0.12 ng/L in healthy people, which is approximately 10 times less than the concentration detectable by standard high-sensitivity methods. Due to such high sensitivity, troponin I was detected in 96.8% of completely healthy people [11].

High sensitivity of new (high-sensitivity and ultra-sensitive) test systems allowed for the development of MI early-diagnostic algorithms. During the first 1–3 h, low levels of cTnT and cTnI, which used to be “invisible” for moderately sensitive test systems, became clearly identifiable by modern immunoassays [1,12,13]. Moreover, it became possible to demonstrate that cardiac troponins in low concentrations are present in oral fluid and urine [14,15,16,17,18,19]. This is a new and very promising direction in the non-invasive diagnosis of both the cardiovascular diseases and pathologies that cause myocardial damage [20].

It is well understood that, apart from MI, the serum levels of cTnT and cTnI grow in many pathological (arterial hypertension, pulmonary artery thromboembolism (PATE), atrial fibrillation, heart failure, chronic renal failure (CRF), chronic obstructive pulmonary disease (COPD), etc.) and physiological (physical exercise, psycho-emotional stresses) conditions as well as the cardiotoxicity of drugs [21,22,23,24,25,26,27,28,29,30].

Taking into consideration the mechanisms of the increase in cardiac troponins, the three groups of the causes for cardiac troponins’ increase can be identified (Table 1): (1) increase in cTnT and cTnI levels associated with myocardial injury in primary cardiac disease, (2) increase in cTnT and cTnI levels associated with myocardial injury in non-cardiac diseases, and (3) increase in cTnT and cTnI levels associated with preanalytical and analytical factors. In the latter case, the increase in cTnT and cTnI levels takes place without myocardial injury and is conditioned upon the influence of physical and chemical (hemolysis, lipemia, presence of clots in a sample, etc.) or biological (presence of heterophile antibodies, increase in the level of bilirubin, alkaline phosphatase, rheumatoid factor) factors on the result of the laboratory test [31,32,33,34,35,36,37].

The increase in cTnT and cTnI associated with analytical and preanalytical factors not only bears no diagnostic or prognostic value but also can have an extremely adverse impact on treatment and the diagnostic process. The most significant contribution, with regard to the change of concentration (degree of increase) of cardiac troponins, is made by heterophile antibodies. This paper consistently considers the causes and mechanisms of formation of heterophile antibodies and shows their influence on the concentrations of cTnT and cTnI. The methods for detection and control of this interference are discussed as well.

## 2. Influence of Heterophile Antibodies on the Concentrations of cTnT and cTnI: Clinical Data

Heterophile antibodies are endogenous antibodies in human serum/plasma that can interfere with immunoassays leading to false elevation or (rarely) false depression of measured values [38,39,40,41,42]. The incidence of heterophile antibodies is extremely variable and amounts to 0.1–3% in the general population [38,39,40]. The main causes of formation of heterophile antibodies are contact with domestic and wild animals, blood transfusion, autoimmune diseases, hematologic malignancies, dialysis, and pregnancy [40]. Heterophile antibodies have recently become an object of active attention, because they significantly affect the results of laboratory test values. At the same time, such an interference relates to practically all medical fields and is by no means limited to cardiology. In their article “When lab tests lie. Heterophile antibodies”, A. Morton notes that heterophile antibodies cause a number of problems in diagnostics of many diseases that imply the use of immunochemical (immunoenzymometric, immunochemiluminiscent, immunofluorescence, radioimmune) methods for biomarkers detection [40]. Heterophile antibodies may influence a wide range of laboratory tests, resulting in false elevation of tumor markers, hormone levels, MI markers, therapeutic-drug-monitoring results, etc. [39,40,41]. 

The first clinical case of a cTnI false-positive result generated by the influence of heterophile antibodies was described in 1998 by T. Fitzmaurice et al. [42]. A 69-year-old patient underwent an infrarenal aneurysm surgery. The concentration of cTnI after the surgery equaled 106 μg/L, while the norm is 0.5 μg/L. In the 5 h after the first test, the concentration of cTnI increased approximately 1.5 times and reached the value of 146 μg/L, which made the physicians think of an ischemic myocardial injury. At the same time, the data of clinical and functional methods, including an ECG, did not confirm the development of myocardial ischemia, and the concentration of another marker—creatine phosphokinase MB isoform (CPK-MB)—was within normal limits as well (2.9 μg/L). After treating this sample with a special solution—the heterophile antibody blocking agent (HABA)—the concentration of cTnI decreased to 1.5 μg/L, though it was still above normal. When the patient’s blood specimens (the untreated sample and the sample treated with the HABA) were investigated using other immunochemical test systems for detection of cTnI and cTnT, the detected concentrations of these biomarkers were normal.

Two years later, S. Kazmierczak et al. found a false-positive increase in cTnT and cTnI in a 75-year-old woman after a surgery. It is noteworthy that during the period of their stay at the hospital, the levels of cTnT and cTnI repeatedly increased, reaching 40 μg/L, and then sharply dropped, which is indirectly indicative of the influence of some other (non-ischemic) factors on the results. Besides, during the incubation of blood samples with nonimmune mouse serum, the levels of cTnI and cTnT decreased in all the samples obtained from the patient approximately two times [43].

K. Yeo et al. analyzed 200 serum samples with positive values of cTnI. After adding the HABA, it turned out that four blood samples had false-positive results, i.e., the share of false-positive results was 2%. The concentration of cTnI in untreated blood samples and blood samples treated with the HABA changed between 2 and 70 times. Testing of the same blood samples using another test system for detection of cTnI did not demonstrate any signs of heterophile antibody interference [44]. D. Uettwiller-Geiger et al. investigated the levels of cTnI in 101 samples of patient serum via the Access AccuTnI test system (Beckman Coulter) and detected the interference of heterophile antibodies in two samples, which is 2%. The incubation of these samples with the HABA efficiently reduced the concentration of cTnI to reference values [45].

G. White et al. described a case of false-positive elevation of cTnT in a 46-year-old man, who was seeking medical attention and complaining of pains in the chest and left arm. The concentration of cTnT in whole blood measured using the quantitative express test Roche CARDIAC T was equal to 0.59 μg/L, which was 5.9 times higher than the upper reference limit (0.1 μg/L). Due to the suspected MI, the patient had to undergo coronary angiography, which appeared normal. The analysis of cTnT concentration in the serum of the patient via another analyzer (Roche T STAT) also did not show any elevated values. After the treatment of whole blood with normal mouse serum (Sigma-Aldrich Co., St. Louis, MO, USA), the cTnT level dropped to the reference limit (0.1 μg/L) [46]. Italian researchers led by M. Cassin described two cases of the cTnI false-positive increase caused by heterophile antibodies at once (Dade Behring RXL Dimension). In the first case, a 64-year-old woman with PATE had constantly variating elevated levels of cTnI (between 23 μg/L to 0.43 μg/L) in comparison with the reference range (0.13 μg/L). Although the physicians suspected a myocardial injury, which is often typical of PATE [47,48], they were confused by the fact that the ECG data and the CPK-MB values were absolutely normal, therefore, the physicians assumed interference was taking place. After the patient’s sample was treated with the HABA, the cTnI level turned out to be lower than the upper-control limit (<0.13 μg/L). The second patient admitted to the emergency department with chest pains also had fluctuations of their cTnI level (Dade Behring RXL Dimension) between 0.19 and 0.36 μg/L, which made the medical team think of MI. However, the normal results of an ECG and the levels of CPK-MB still confused the physicians during the final diagnosis. The treatment of samples with the HABA solution led to the normalization of cTnI values and helped to exclude the diagnosis of MI [49].

Knoblock et al. described a clinical case of false-positive elevation of cTnI in a 53-year-old patient, who had an MI in the past. They were hospitalized for complaints of chest pain and myocardial reinfarction was suspected. The concentration of cTnI (Abbott AxSYM cTnI) in their serum at admission was 6.2 μg/L, which was significantly higher than the reference value (0.4 μg/L). When blood was taken 8 and 24 h from the moment of admission, the levels of cTnI were 5.5 and 5.1 μg/L, respectively. However, the ECG data and the levels of CPK and CPK-MB were within normal limits. A dipyridamole test also showed no signs of myocardial ischemia, and, according to the data of echocardiography, the ejection fraction complied with the norm. Later, this patient applied to the emergency department several times with similar complaints. The results of a coronarography did not detect any abnormalities of the lumen of coronary heart vessels. In general, within 3 months, this patient had 16 positive results of cTnI using an AxSYM analyzer. Due to the constantly elevated levels of cTnI not corresponding to the clinical picture, the data of the clinical and functional investigation methods, and the laboratory results of the other cardiac markers, a false-positive result was suspected. When detecting cTnT (Roche Elecsys 2010) and cTnI using another test system (Dade-Behring Dimension RxL), the results turned out to be significantly lower than the LoD for these test systems. It is noteworthy that, as opposed to other cases, addition of the HABA in the case of this patient led to an even greater increase in the concentration of cTnI. To eliminate interfering antibodies, the researchers passed the serum sample through a protein A immobilized column (Sigma-Aldrich Co.), after which the concentration of cTnI decreased from 7.9 μg/L to 0.2 μg/L [50].

W. Kim et al. conducted a survey, in the course of which they detected 25 cases of false-positive elevation of cTnI (Dade Behring RXL Dimension) caused by heterophile antibodies of class G (IgG). These patients suffered from different diseases, which in the opinion of the researchers could induce the formation of heterophile antibodies: endocrine disorders, recent surgeries, heart diseases, CRF, lung troubles (COPD, PATE), gastro-intestinal diseases, cancer, and connective-tissue diseases. Then, the researchers estimated the efficiency of neutralization of interfering effect of heterophile antibodies using the HABA. In 9 of 13 serum samples, the action of the HABA was effective and resulted in a significant decrease in cTnI levels. At the same time, in four serum samples, the researchers failed to normalize the values of cTnI using the HABA, as well as by using mouse serum containing immunoglobulins IgG1/IgG2a, on the basis of which the authors assumed the presence of heterophile antibodies specific to the analysis components different from heterophile antibodies or mouse immunoglobulin [51].

M. Zaninotto et al. registered a case of cTnI false-positive increase in a 29-year-old woman having a history of infectious myocarditis. The concentrations of cTnI, determined with the help of Dade–Behring RxL, were significantly elevated all the time and varied between 6.0 μg/L and 12.2 μg/L, while the norm is up to 0.15 μg/L. Having suspected a falsely elevated value, due to the inconsistency between the laboratory data and the clinical picture, the researchers measured the levels of cTnI using other test systems; the concentration in this case turned out to be diagnostically insignificant. Having assumed the influence of heterophile antibodies, the researchers treated the serum sample with the HABA, which resulted in a sharp drop of cTnI concentration from 7.73 μg/L to 0.15 μg/L [52].

S. Fleming et al. examined the incidence of false-positive values of cTnI (Access AccuTnI). For that purpose, the researchers systematically incubated all the serum samples that exceeded the diagnostic threshold. The total incidence of falsely elevated troponins conditioned upon the influence of heterophile antibodies equaled 3.1% (95% CI, from 2 to 4.4%) in the general population and 14.8% (95% CI, from 9.9 to 20.9%) in patients with diagnostically significant values of cTnI [53].

Investigating the levels of cTnI (Dade-Behring RXL Dimension) in the serum of 60 patients with legionellosis, M. Garcia-Mancebo et al. discovered that in 47% of cases the concentration of cTnI exceeded the reference limit (0.1 μg/L). The authors noted the following remarkable observation: the regression analysis between the serum antibody titer to Legionella pneumophila and the concentration of cTnI in a sample with interference demonstrated reliable correlation (r = 0.72; *p* < 0.05) [54].

C. Bionda et al. reported a case of false-positive elevation of the cTnI level (Dade–Behring X-Pand) in a patient hospitalized for asthenia, exophthalmos, and sinus tachycardia. Although the concentration of cTnI was significantly elevated, the data of the clinical picture and an ECG did not correspond to ischemic myocardial injury. After the blood sample was treated with the HABA, the concentration of cTnI dropped from 11.4 μg/L to 0.08 μg/L [55].

Y. Zhu et al. found false-positive cTnI in an 88-year-old patient sent to the emergency department for aspiration pneumonia. The level of cTnI measured with the Siemens ADVIA Centaur test system was 19.99 μg/L, while the norm is up to 0.06 μg/L. Due to the inconsistency between cTnI values and the clinical picture, the decision was taken to repeat the investigation of the blood sample using another test system, Abbott i-STAT cTnI. As anticipated, the concentration of cTnI turned out to be lower than the upper reference limit (<0.09 μg/L). After incubation of the serum sample obtained from this patient, the level of cTnI measured via the Siemens ADVIA Centaur test system significantly decreased to 0.03 μg/L [56].

S. Ghali et al. described a clinical case of falsely elevated cTnI in a 74-year-old patient, admitted to the emergency department with a clinical picture resembling the MI: chest pain and increasing dyspnea [57]. The level of cTnI (Beckman Coulter Access AccuTnI) was significantly elevated to 77.28 μg/L, while the reference range is 0.00–0.04 μg/L for this test system. At the same time, the ECG data did not correspond to myocardial ischemia but were indicative of the right bundle-branch block: hypertrophy of the walls of the left heart chambers. Other cardiac markers detected in this patient, as opposed to cTnI, were within the reference limits: myoglobin (50 ng/mL), CPK-MB (5.2 ng/mL), CPK (74 IU/L), and D-dimers (0.33 μg/L). The data of transthoracic echocardiography revealed hypertrophy of the left ventricle, mild diastolic dysfunction, and normal ejection fraction, without any signs of regional contractility disorders. Notwithstanding the fact that these morphological changes of the myocardial hypertrophy can, to some extent, explain the increase in the concentration of cardiac troponins, in this case the increase level turned out to be too significant and could by no means correspond to the patient’s condition. Besides, the level of creatinine reflecting the condition of kidney filtration was also normal, which additionally excluded the reason for the increase in cTnI associated with its elimination from the bloodstream. The levels of cTnI remained elevated during the whole hospitalization period, keeping disproportionality with regard to other cardiac markers (myoglobin, CPK-MB, and CPK), which either stayed normal or were very insignificantly elevated during the whole period. Doubting the results of the cTnI level obtained in their laboratory, the decision was taken to send the patient’s serum samples to another hospital’s laboratory using another immunological method of detecting cTnI (Siemens ADVIA Centaur). The levels of cTnI in the samples obtained from this patient were constantly lower than 0.01 μg/L. Carrying out further investigation to clarify the influence of heterophile antibodies, the researchers added to the patient’s serum 10 various blocking agents manufactured by Scantibodies Laboratory: immunoglobulins of class G (IgG), goat IgG, mouse IgG, rabbit IgG, bovine IgG, Poly Mak 33, Scavenger ALP, AP Mutein, HBR-1, HBR-non murine, and TRU block. The first seven agents listed above are specific agents blocking heterophile antibodies. The last three are agents blocking nonspecific heterophile antibodies. Nine of the ten blocking agents did not influence the resulting level of cTnI. However, adding the nonspecific blocking-agent HBR-1 reduced the result of the troponin by more than 90% of its initial value, which is indicative of successful blockage of heterophile antibodies in this patient. Another interesting observation of the authors was that the hemoglobin-level titer during the whole period of the patient hospitalization was very closely correlated with the concentration of cTnI, which means it actually reflected the influence of heterophile antibodies and could be used as a surrogate marker of the heterophile antibody titer. Thus, in the course of hospital treatment, the patient had a duodenal ulcer hemorrhage caused by anticoagulant therapy, which led to a drop in hemoglobin levels followed by a decrease in cTnI level, caused by the heterophile-antibody-titer drop [57].

The Danish researchers H. Nørlund et al. described a case of cTnI false-positive increase in a 32-year-old pregnant patient, admitted for complaints of chest pain and dyspnea. Application of additional methods of clinical, functional, and laboratory diagnostics allowed for the exclusion of the main possible reasons ‒ MI, PATE, myocarditis, and others. The researchers made an assumption that those changes were connected with pregnancy [58].

J. Nguyen et al. also recently reported an interesting clinical case of cTnI false-positive elevation (Access AccuTnI + 3^TM^) in a 52-year-old patient, who requested medical assistance in the emergency department regarding the complaints of chest pain. The level of cTnI was elevated at admission and remained such (neither increasing or decreasing) during the whole period of in-treatment. At the same time, the data of an ECG and the results of surrogate biomarkers of MI (CPK, CPK-MB;, myoglobin) during the whole hospitalization period did not indicate ischemia and/or myocardial-tissue injury. The patient also underwent echocardiography, a heart catheterization, and a computer tomography, however only insignificant cardiac changes were detected (tricuspid regurgitation of the first degree, insignificant pericardial effusion, and the signs of a non-obstructive lesion in the coronary bed). The medical team expressed their doubts about the result of the laboratory test and sent the patient’s serum sample for cTnI testing to another laboratory, applying the test system Advia Centaur XP TnI-Ultra assay. Troponin in this patient appeared negative. Carrying out further investigation to identify the causes of interference, the researchers diluted the serum sample two-fold, which under normal conditions should have resulted in the proportional drop of the troponin level, however, the values in that case, on the contrary, increased to 6.13 μg/L, which is one of the signs of heterophile-antibody interference. However, at the same time, the adding of the HABA did not lead to the normalization of troponins, and when the serum was tested for heterophile antibodies, they turned out to be negative [59].

L. Manjunath et al. reported a case of possible influence of heterophile antibodies on the concentration of troponin I in a young patient [60]. The patient was admitted to the emergency department with chest discomfort. The level of cTnI at admission was 0.123 μg/L (while the norm is up to 0.055 μg/L) and later rose to 0.124 and 0.213, which is typical of a classical MI. Besides, the version of MI was supported by an adverse lipid profile of the patient: total cholesterol in the fasted state was 235 mg/dL, low-density lipoprotein was 170 mg/dL, high-density lipoprotein was 38 mg/dL, and triglycerides were 124 mg/dL. However, the laboratory data concerning other MI surrogate markers did not detect any signs of myocardial injury that could lead to such a significant increase in troponin I. When taking the history, the medical team found out that the patient was actively participating in sports, and on the day before the admission they had run several kilometers while preparing for a marathon race, which made the researchers think about the influence of physical exercise [61,62]. Nevertheless, in this case it is highly unlikely that the result was distorted only by the impact of physical exercise. Further follow-up of the patient always showed chronic troponinemia, even independently of physical exercises. Therefore, excluding all the factors known, the researchers came to the conclusion of possible influence by heterophile antibodies [60].

There are few research papers about the influence of heterophile antibodies on the concentration of high- and ultra-sensitive troponins [63,64,65,66,67]. S. Baroni et al. described a case of false-positive elevation of high-sensitive troponin I (TNIH Centaur XPT Siemens) in a 52-year-old man admitted with chest pains radiating to the left arm. The physical examination data were normal. An ECG registered sinus rhythm and the absence of significant changes of the ST segment and the T wave. Arterial-blood pressure was elevated to 170/90 mm Hg. The patient’s laboratory values, including creatinine and creatine kinase, were within the reference ranges. Cardiac troponin measured using the standard cTnl-ultra Siemens test (the norm is up to 0.040 μg/L) was negative, both at admission and 3 h afterward (0.012 and 0.008 μg/L respectively). Along with that, the investigation of the blood sample using another high-sensitivity analyzer TNIH Centaur XPT Siemens (the norm is up to 47 ng/L) detected elevated levels of troponin I—129 ng/L and 140 ng/L at admission and after 3 h, respectively. Before that episode, the researchers tested using this kit about 100 healthy patients, and the values of all of them fell within the reference range. In addition to the laboratory examination, the patient also underwent an ECG, echocardiography, and an ECG stress test, which did not detect any signs of myocardial ischemia that could have led to such a remarkable increase in troponins. Later, the physicians continued observations and upon blood collection after 6 and 12 h the elevated levels were also registered with a TNIH Centaur XPT Siemens analyzer (132 ng/L and 128 ng/L respectively), while the analyzer cTnl-ultra Siemens always, on the contrary, showed negative levels. In order to identify the cause of interference, the patient’s serum was serially diluted with a sample of serum with an undetectable troponin concentration, for the purpose of checking the linearity of the results. Serial dilutions demonstrated that the values obtained using the TNIH Centaur XPT kit were non-linear (1:2–55 ng/L; 1:4–33 ng/L; 1:8–21 ng/L; 1:16–12 ng/L; 1:32–9 ng/L), which suggests the presence of an interfering substance in the patient’s sample [63].

N. Lacusik et al. presented a clinical case of false-positive cTnI in a 53-year-old woman, hospitalized via the emergency department for complaints of retrosternal discomfort. Upon examination, no peculiarities were found, an ECG showed no ischemic changes, and echocardiography did not detect any areas of contractility disorders, but the cTnI value was equal to 1359 ng/L, which led to the decision to perform a coronarography. The results of the intervention showed borderline 70% stenosis of the left circumflex artery in proximal segment, followed by a drug-eluting stent insertion. The patient was dismissed on the seventh day without complications. Then, in 3 weeks, rehospitalization was required due to complaints of retrosternal pain. An ECG showed no ischemic changes, however, because of a significant increase in cTnI, an emergency coronarography was performed. No stent thrombosis, restenosis, or new stenoses in other arteries were identified. During the whole hospitalization period, the values of cTnI remained elevated. Then, the patient was transferred to the rehabilitation department, where they also complained of retrosternal pains, and the concentration of cTnI was equal to 1111 ng/L. Several repeated measurements showed that the level remained similar. Taking into consideration this fact, the researchers suspected the presence of heterophile antibodies [68].

L. G. Santos et al. wrote about a 57-year-old patient with a stable elevation of cTnI level after a clinically suspected myocarditis. The patient was hospitalized via the emergency department for complaints of retrosternal pain radiating to the left upper extremity, with a duration of more than 3 h. Clinical examination showed no peculiarities, except for the fact that the patient reported they had been sick with the flu two weeks before the hospitalization. An ECG registered negative T waves in V1 and V2 leads and biphasic T waves in leads V3–V5. An increase in creatine kinase, to 380 IU/L (the reference value is 10–195 IU/L), and in cTnI, to 6.24 ng/mL (less than 0.04 ng/mL), was detected. The patient underwent a coronarography that detected no obstructive lesion of coronary arteries. A myopericarditis was diagnosed, and the corresponding treatment was initiated, which was followed by a hospital discharge. In 4 weeks, the patient was seeking medical attention again with similar complaints. The level of cTnI was 10.46 ng/mL. A repeated coronarography was performed and it detected no abnormalities. On the fourth day of hospitalization, the level of cTnI was 26.81 ng/mL. Due to the increase in the concentration of cardiac troponin without the visible clinical picture of MI, the researchers assumed the presence of heterophile antibodies circulating in the blood, which was proven later [39].

**Figure 1:**
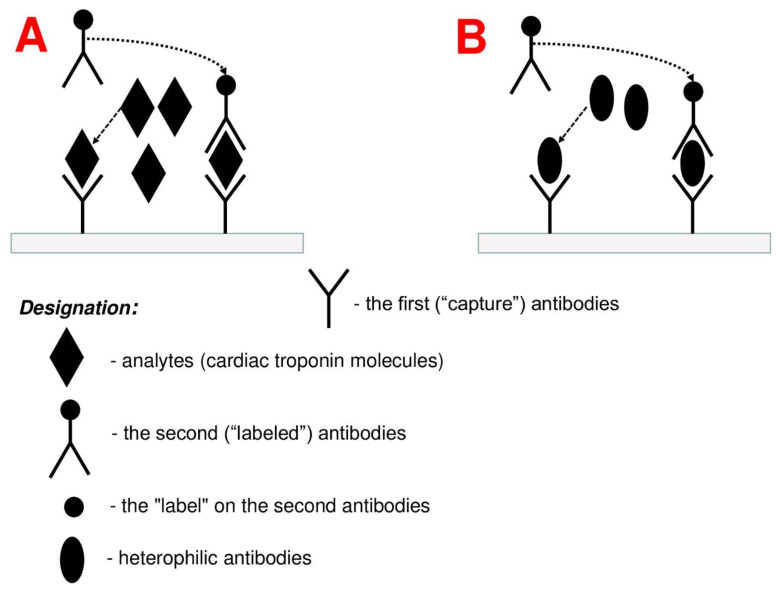
Mechanism of the influence of heterophile antibodies on the concentration of cardiac troponins. Figure description: (**A**) **Absence of interference.** Classical immunoassay includes two stages. At the first stage, the analytes (molecules of cardiac troponins) being released in case of myocardial injury interact with the first (“capture”) antibodies, which results in the formation of an antigen–antibody complex. Then, the second (“labeled”) antibodies bind with this complex, which leads to the formation of a “sandwich-type” immune complex. The label on the second antibodies causes the generation of a signal, the level of which is directly proportional to the quantity of the antigen–antibody complexes formed at the first stage, i.e., the concentration of cardiac troponin molecules in the examined biological fluid sample. (**B**) **Presence of interference.** Heterophile antibodies can unspecifically bind with capture antibodies at the first stage in the absence of the analytes of interest (molecules of cardiac troponins) in biological fluid and lead, therefore, to false-positive results.

Taking into consideration what was said above, almost all test systems used for detection of cTnT and cTnI are subject to the influence of heterophile antibodies. According to the data of some researchers, a certain test system is weakly influenced by heterophile antibodies, while other researchers claim that the same test system can often show false-positive results. Therefore, it is not possible yet to identify the most reliable test system that is influenced by heterophile antibodies to the least extent. At the same time, nowadays there is no 100% reliable method that could ensure neutralization of the heterophile-antibodies effect. The main mechanisms of influence of heterophilic antibodies are their ability to interact with diagnostic antibodies (antibodies against cTnT and cTnI) (Figure 1).

D. Herman et al. suppose that the influence of heterophile antibodies on levels of cardiac troponins can depend upon the type of a sample being examined. For troponin testing, the following types of samples may be used, depending on the type of the applied tube that can contain additives of different compositions: serum-separator tube—with a red or gold top (without additive), plasma-preparation tube—with a green top (contains anticoagulant heparin), and whole-blood tube—with a purple top (contains anticoagulant EDTA, besides, this tube can be centrifuged for preparation of plasma). The most frequently used tubes for quantitative detection of cardiac troponins are serum-separator tubes and heparinized tubes. The researchers have found that the examination of blood collected from the same patient using different tubes can give different troponin levels. It is assumed that the possible difference between the samples of serum (red-top tube) and heparinized plasma (green-top tube) may appear if antibodies are affected by heparin. Since a molecule of cTnI has a high positive charge, it will attract negatively charged molecules such as heparin, which, in turn, can hinder the interaction of antibodies and antigens, thus reducing their interfering influence [69]. For confirmation of this phenomenon, additional studies are needed.

## 3. Methods for Detection and Control of Heterophile Antibodies

Blocking agents are used to control heterophile antibodies. An additional and rather effective way of controlling heterophile antibodies is the several-fold dilution of samples of patient serum and a check of the linearity (proportionality) of the results: i.e., under normal conditions, if a patient’s sample with the determined concentration is diluted two-fold, the level of troponins should also decrease approximately two times. In the case of four-fold dilution, the level of troponins should decrease four times, and so on. For a complete check of the linearity, it is optimal to consider the results of 3–4 serial dilutions. If the level of troponins in serum that has been diluted several-fold decreased disproportionally or even increased, the influence of heterophile antibodies should be suspected. Other methods for detection of heterophile antibodies are not supported with a sufficient evidence base, as they did not demonstrate reproducible results.

According to clinical studies, the prevalence of false-positive results due to the influence of heterophilic antibodies is 2–47% [41,42,43,44,45,53,54] (Table 2). The reason for the wide range of false-positive results is definitively unknown. Hypothetically, this may be due to the cause of the formation of heterophilic antibodies (for example, in patients with legionellosis, the frequency of false-positive results is very high [54]), the method of determining cardiac troponins, the characteristics of the examined population, etc. There are very few studies devoted to this issue. More research is needed to clarify this aspect. This will help identify people, who are most at risk of forming false-positive results due to the influence of heterophilic antibodies, justify the need for routine use of HABA solutions.

## 4. Conclusions

Thus, heterophile antibodies are an uncommon-yet-significant cause of false-positive concentrations of troponins. Apparently, heterophile antibodies influence all known troponin test systems. The mechanisms of influence on the results are associated with their interference at the stage of immunological interaction between the diagnostic antibodies against the troponin molecule of interest and the antigen (the troponin molecule of interest). The main and the most reliable methods for their detection are blocking agents, the addition of which leads to a decrease in or complete normalization of concentrations. In the absence of this agent in the laboratory, the indirect method for heterophile detection can be applied, which involves serial dilution of the sample. If troponin levels in the sample change in the manner significantly disproportionate to the dilution, the presence of heterophile antibodies may be suspected.

## Figures and Tables

**Table 1:** Three groups of the causes for cardiac troponins’ increase.

Increase in cTnT and cTnI Level Associated with the Myocardial Injury in Primary Cardiac Disease	Increase in cTnT and cTnI Associated with Myocardial Injury in Non-Cardiac Diseases	Increase in cTnT and cTnI Associated with Preanalytical and Analytical Factors
- MI, - Cardiomyopathy - Acute and chronic myocarditis - Takotsubo syndrome - Arrhythmias - Heart surgery - Cardiac contusion - Infiltrative pathologies (amyloidosis of the heart, etc.)	- CRF - COPD - Cardiotoxicity of drugs - Sepsis - PATE - Physical exercise, psycho-emotional stresses	- Hemolysis - Lipemia - Bilirubin - Clots - Heterophile antibodies - Alkaline phosphatase - Rheumatoid factor.

Abbreviations: cTnT—cardiac troponin T, cTnI—cardiac troponin I, MI—myocardial infarction, COPD—chronic obstructive pulmonary disease, CRF—chronic renal failure, PATE—pulmonary artery thromboembolism.

**Table 2:** The prevalence of false-positive results due to the influence of heterophilic antibodies.

Number of Samples, Diagnosis	Frequency of False-Positive break/Troponins Associated with Heterophilic Antibodies, %	Source
n = 200, healthy people	2	[44]
n = 101, healthy people	2	[45]
n = 767, healthy people	3.1	[53]
n = 60, legionellosis	47	[54]

## Data Availability

Not applicable.
